# Role of ferroptosis-related genes in Stanford type a aortic dissection and identification of key genes: new insights from bioinformatic analysis

**DOI:** 10.1080/21655979.2021.1988840

**Published:** 2021-11-30

**Authors:** Hua-Xi Zou, Bai-Quan Qiu, Song-Qing Lai, Huang Huang, Xue-Liang Zhou, Cheng-Wu Gong, Li-Jun Wang, Ming-Ming Yuan, an-Di He, Ji-Chun Liu

**Affiliations:** a Department of Cardiothoracic Surgery, The Second Affiliated Hospital, Nanchang University, Nanchang, Jiangxi, China; b Department of Cardiothoracic Surgery, The First Affiliated Hospital, Nanchang University, Nanchang, Jiangxi, China

**Keywords:** Ferroptosis, Stanford type a aortic dissection, key genes, database, bioinformatics analysis

## Abstract

Stanford type A aortic dissection (TAAD) is one of the most dangerous vascular diseases worldwide, and the mechanisms of its development remain unclear. Further molecular pathology studies may contribute to a comprehensive understanding of TAAD and provide new insights into diagnostic markers and potential therapeutic targets. Recent studies have identified that ferroptosis, a form of cell death, may play a previously unrecognized role in influencing the development of TAAD. In this study, we explored the pathological role of ferroptosis in TAAD by performing bioinformatics analyses. Gene set enrichment analysis (GSEA) showed that the ferroptosis-related gene (FRG) set was significantly different between normal and TAAD aortic samples at an overall level (*p* < 0.001). Further Gene Ontology (GO) and Kyoto Encyclopedia of Genes and Genomes (KEGG) enrichment analyses explored the potential functions and pathways of FRG in TAAD. We further identified six key genes (CA9, HMOX1, IL6, CDKN1A, HIF1A, MYC) from differentially expressed FRGs in TAAD by constructing a protein–protein interaction (PPI) network, all key genes were upregulated in TAAD. Four of the key genes (CA9, IL6, CDKN1A, and HIF1A) were demonstrated to be correlated with cigarette smoke extract-induced ferroptosis in aortic vascular smooth muscle cells. These results suggest that ferroptosis is one of the essential pathological processes in the development of TAAD, and some FRGs affect TAAD development by mediating cellular ferroptosis, which provides deepening insights into the molecular mechanisms and potential therapeutic targets of TAAD.

## Introduction

Aortic dissection (AD) is a life-threatening condition with high morbidity and mortality, caused by a tear in the intimal layer of the aorta or bleeding within the aortic wall [1]. Stanford type A aortic dissection (TAAD) is one of the most dangerous types of AD. Although the in-hospital mortality rate of TAAD has decreased with innovations and improvements in imaging and surgical techniques, its diagnosis and treatment remain a challenge [2,3].

Attaining an essential understanding of the molecular and pathological mechanism may provide new insights into therapeutic targets for TAAD. Smooth muscle cell alteration, extracellular matrix degradation, and inflammatory cell infiltration are thought to be the main pathological mechanisms in the development of AD [4]. Vascular smooth muscle cells (VSMCs) are the major cellular component of the aortic wall and play a central role in maintaining aortic function and homeostasis [5,6]. Various factors and types of stress induce alterations in VSMCs, such as abnormal differentiation, migration, and apoptosis, which impair the structural and functional integrity of VSMCs, leading to aortic degeneration and ultimately biomechanical failure, which is an important pathological mechanism for the formation of TAAD [4,6,7].

Ferroptosis is an iron-reliant and reactive oxygen species (ROS)-dependent regulated cell death, which is caused by lipid peroxidation and characterized by the ruptured mitochondrial outer membranes, decreased or vanished mitochondrial cristae, and condensed mitochondrial membranes [8–10]. Ferroptosis is a complex process regulated by various mechanisms with great potential in clinical therapies, and it has been demonstrated to be an important therapeutic target in cancer and cardiovascular disease [11–13]. Although trace element measurements have revealed elevated Fe content in AD tissues [14], the relationship between ferroptosis and AD and the regulatory mechanisms are still poorly defined. Recently, a study revealed that ferroptosis plays an important role in cigarette smoke extract (CSE)-induced VSMC cytotoxicity through intracellular GSH depletion [15]. Cigarette smoking is a major risk factor for AD [16–18], and we speculate that there may be some correlation between ferroptosis and the development of TAAD.

Here, to determine whether and how ferroptosis contributes to the development of TAAD, we performed a bioinformatics analysis using datasets from public databases. We analyzed the relationship between FRGs and the pathogenesis of TAAD, explored the potential functional mechanism and key genes of FRGs in TAAD, and performed a primary validation through vitro experiments, which contributes to further explore the pathological mechanisms and therapeutic strategies of TAAD.

## Materials and methods

### Data collection

The RNA-seq dataset GSE153434 and the microarray dataset GSE52093 were downloaded from the Gene Expression Omnibus (GEO) database of the NCBI database (https://www.ncbi.nlm.nih.gov/geo/). The dataset GSE153434, which was performed on the GPL20795 (Illumina HiSeq X Ten Homo sapiens) platform, contains whole transcriptome expression data from 10 TAAD ascending aorta samples and 10 normal ascending aorta samples and was used to screen for differentially expressed genes (DEGs). The GSE52093 dataset, which was performed on the GPL10558 (Illumina HumanHT-12 V4.0 expression bead chip) platform, contains whole-transcriptome expression data from 5 TAAD ascending aorta samples and seven normal ascending aorta samples and was used to verify the hub genes. After ID conversion, the average expression value was taken as the gene expression value when multiple probes corresponded to a gene. The RNA-seq data were normalized based on dispersion, and the microarray data were log2 transformed and quantile normalized.

### Identification of DEGs and DEFRGs

DEGs were identified using the DESeq2 package (version 3.11.0) in R software (version 3.6.1), and *p*-values were adjusted using the Benjamini and Hochberg method [19]. A false discovery rate (FDR) <0.05 and |log2FC| ≥1 were defined as the thresholds for DEG selection.

FRGs were obtained from FerrDb [20] (https://www.zhounan.org/ferrdb) and GeneCards [21] (https://www.genecards.org). After deduplication of genes, the merged FRG set contained 442 FRGs, as listed in **Table S1**. Finally, 59 overlapping DEFRGs were selected using the VennDiagram package (1.6.20) in R software.

### Gene set enrichment analysis

GSEA using the GSEA software (software.broadinstitute.org/gsea/index.jsp) was performed to observe the overall correlation between ferroptosis and TAAD. The ferroptosis-related gene set contains 442 FRGs obtained as mentioned. Genes in GSE153434 were scored and ranked by expression value to calculate the enrichment score (ES) and core genes. FDR < 0.25 and normal *p* < 0.05 were considered significant in our study.

### GO and KEGG enrichment analyses

The DEFRGs identified were subjected to GO and KEGG enrichment analyses using the clusterProfiler package (version 3.12.0) in R software. The results with *p*  <  0.05 were considered significantly enriched by DEFRGs.

### PPI network and identification of key modules and hub genes

The STRING database (https://string-db.org/) and Cytoscape software (version 3.8.2) were used to establish and visualize a PPI network of DEFRGs. Three functional modules were identified by the Cytoscape plugin MCODE (the parameters were set to default: degree cutoff = 2, node score cutoff = 0.2, K-core = 2 and max depth = 100). Another plugin, Cytohubba, was used to identify hub genes. The built-in MCC algorithm of Cytohubba assigned a value to each gene in the PPI network and ranked these genes by values. The top 10 genes were significant and were regarded as hub genes.

### Validation of hub genes in GSE52093

The microarray dataset GSE52093 was used as an independent dataset for validation. After data preprocessing as described previously, the expression data of 10 hub genes were extracted and groups were compared using the Wilcoxon test. The results with *p* < 0.05 were considered statistically significant. Receiver operating characteristic (ROC) curve analyses were performed using the Hiplot software (version 0.1.0) to determine the sensitivity and specificity of the 10 hub genes, quantified by the area under the ROC curve (AUC). Genes with AUC > 0.7 were considered to have diagnostic value.

### Immune infiltration analyses

To estimate the proportion of infiltrating immune cells, normalized gene expression data of GSE153434 and GSE52093 were submitted to Hiplot software (version 0.1.0) and the online analysis tool ImmuCellAI [22] (https://bioinfo.life.hust.edu.cn/web/ImmuCellAI/). The proportion of infiltrating immune cells and the infiltration score were calculated with the CIBERSORT and ssGSEA algorithms.The results with *p* < 0.05 were defined as statistically significant differences.

### Cell culture and reagents

The A7r5 (rat thoracic aortic VSMC) cell line was obtained from Cell Bank/Stem Cell Bank (Chinese Academy of Sciences, China) and cultured in high-glucose Dulbecco’s modified Eagle’s medium (H-DMEM) (HyClone, GE Healthcare Life Sciences, USA), supplemented with 10% fetal bovine serum (FBS) (Gibco, Thermo Fisher Scientific, USA). Cells were incubated at 37°C under standard conditions (5% CO_2_, 95% humidity, 21% O_2_ concentration).

CSE was freshly prepared for each experiment as previously described [23]. In brief, mainstream smoke from two cigarettes (Marlboro brand) was bubbled through 20 ml of culture medium. After adjustment of the pH to 7.4, CSE was filtered through a 0.22 µm filter for sterilization and then standardized by measuring the absorbance at 320 nm. The solution was considered to be 100% CSE and was diluted with culture medium before use. Ferrostatin-1 (Fer-1, GC10380) was purchased from Glpbio Technology Inc. (Montclair, CA, USA) and dissolved in dimethyl sulfoxide (DMSO). In cell culture experiments, Fer-1 was treated 1 h before CSE treatment at a concentration of 5 μM.

### Cell viability assay

CCK8 assay was carried out to determine cell viability. A7r5 cells were seeded in 96-well plates at a density of 5 × 10^3^ cells/well with 6 replicate wells in each group and incubated in H-DMEM for 24 h. Then, the cells were treated with reagents for the indicated times. Finally, 20 µl of CCK-8 reagent was added to each well for another 1 h of incubation following the manufacturer’s instructions(GK10001, Glpbio, USA). The optical density (OD) values were measured at 450 nm using a Multiskan FC microplate reader (ThermoFisher Scientific, USA). Ordinary one-way ANOVA was used to analyze the differences between groups, and *p* < 0.05 was considered statistically significant.

### Quantitative reverse real-time PCR (qRT-PCR)

Total RNA was extracted from the cells using TRIzol reagent (Invitrogen, USA), and the quality and yield of RNA were evaluated to confirm that the isolated RNA can be used to assess mRNA expression. Total RNA was reverse transcribed into cDNA using RevertAid MM (ThermoFisher Scientific, USA). Gene expression levels were quantified by Power SYBR Green PCR Master Mix (ThermoFisher Scientific, USA) using an Applied Biosystems StepOnePlus Real-Time PCR System (ThermoFisher Scientific, USA). The mRNA expression levels were normalized by ACTB (B661202, Sangon Biotech, Shanghai, China) and were calculated according to the 2− ΔΔCT method [44]. Multiple independent samples were used (N =  3). Ordinary one-way ANOVA was used to analyze the differences between groups, and *p* < 0.05 was considered statistically significant. All primers were designed and synthesized by Sangon Biotech Co. Ltd. (Shanghai, China). Detailed primer information can be found in **Table S2**.

## Results

### Overall study protocol

**Figure 1.**
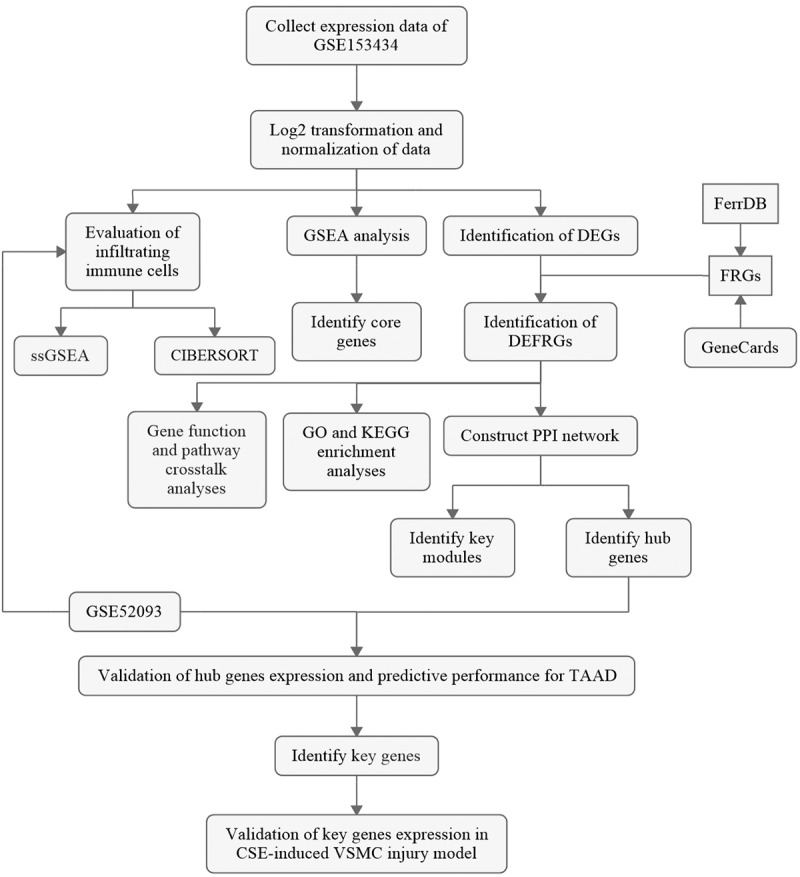
The overall protocol of this study

DEG, differentially expressed gene; FRG, ferroptosis-related gene; DEFRG, differentially expressed FRG; PPI, protein–protein interaction; CSE, cigarette smoke extract; VSMC, vascular smooth muscle cell.

The overall flowchart of the study is summarized in Figure 1.

### Relationship between ferroptosis and TAAD

**Figure 2.**
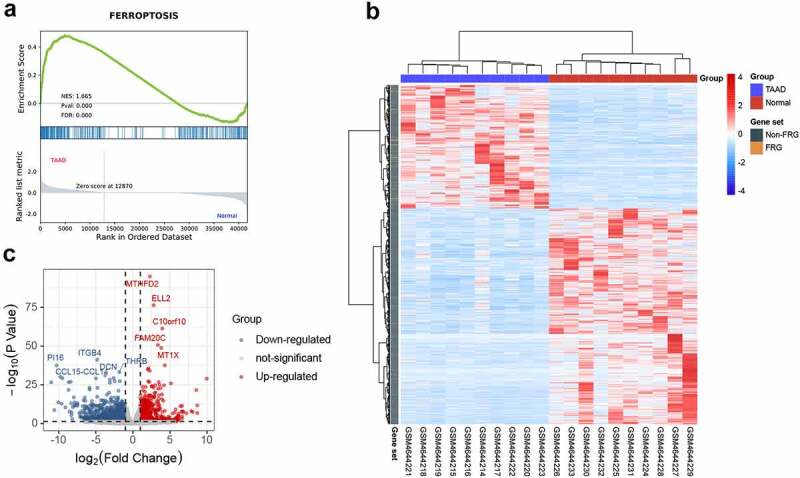
GSEA and identification of differentially expressed genes

GSEA is a conventional approach to identify pathways related to gene expression. To evaluate the involvement of the ferroptosis pathway in the development of TAAD, we performed GSEA of the ferroptosis-related gene set. The GSEA result, as shown in Figure 2a, indicates that the ferroptosis-related gene set was significantly enriched at a nominal *p* value <0.001 (FDR < 25%) in the TAAD samples, and mostly upregulated, which revealed an overall significant correlation between ferroptosis and TAAD. A total of 153 core genes were identified by GSEA, as listed in **Table S3**.

(A) Analysis of relationship between ferroptosis and TAAD by GSEA in GSE153434. (B) Clustered heatmap of DEGs in GSE153434 (FDR < 0.05 and |log2FC| ≥ 1). (C) Volcano plot of DEGs in GSE153434 (FDR < 0.05 and |log2FC| ≥ 1). DEG, differentially expressed gene.

### Identification of DEGs and DEFRGs

The clustered heatmap of DEGs revealed that gene expression between the TAAD and normal samples was distinct (Figure 2b). A total of 1728 DEGs were identified from GSE153434, of which 631 were upregulated and 1097 were downregulated (Figure 2c), as listed in **Table S4**.

**Figure 3.**
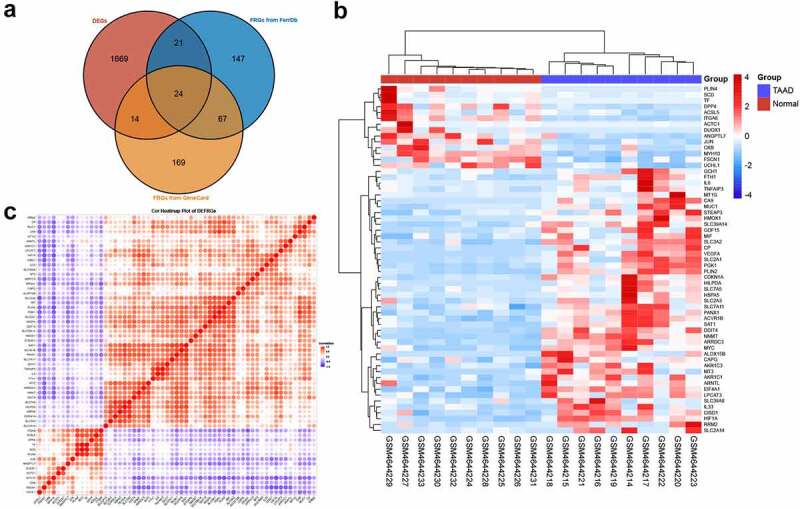
Identification of differentially expressed ferroptosis-related genes

Since the GSEA results showed a tight relationship between ferroptosis and TAAD, we further identified DEFRGs. After deduplication of genes, a total of 442 FRGs were identified from FerrDb and GeneCards. We overlapped FRGs with the DEGs in GSE153434 and selected 59 overlapped DEFRGs for further analyses (Figure 3a), all the DEFRGs listed in **Table S5**. The clustered heatmap and correlation heatmap based on the Ward. D2 algorithm showed the expression differences of 59 DEFRGs between TAAD and normal samples, as well as the correlation between DEFRGs (Figures 3B **and 3 C**).

(A) Venn diagram showing the overlap of genes between DEGs in GSE153434 and FRGs in the FerrDB and GeneCard databases, * = selected DEFRG set. (B) Clustered heatmap of DEFRGs in GSE153434. (C) Correlation heatmap of DEFRGs in GSE153434, correlation coefficients are plotted with negative correlation (blue) and positive correlation (red). DEG, differentially expressed gene; DEFRG, differentially expressed ferroptosis-related gene.

### GO and KEGG enrichment analyses

**Figure 4.**
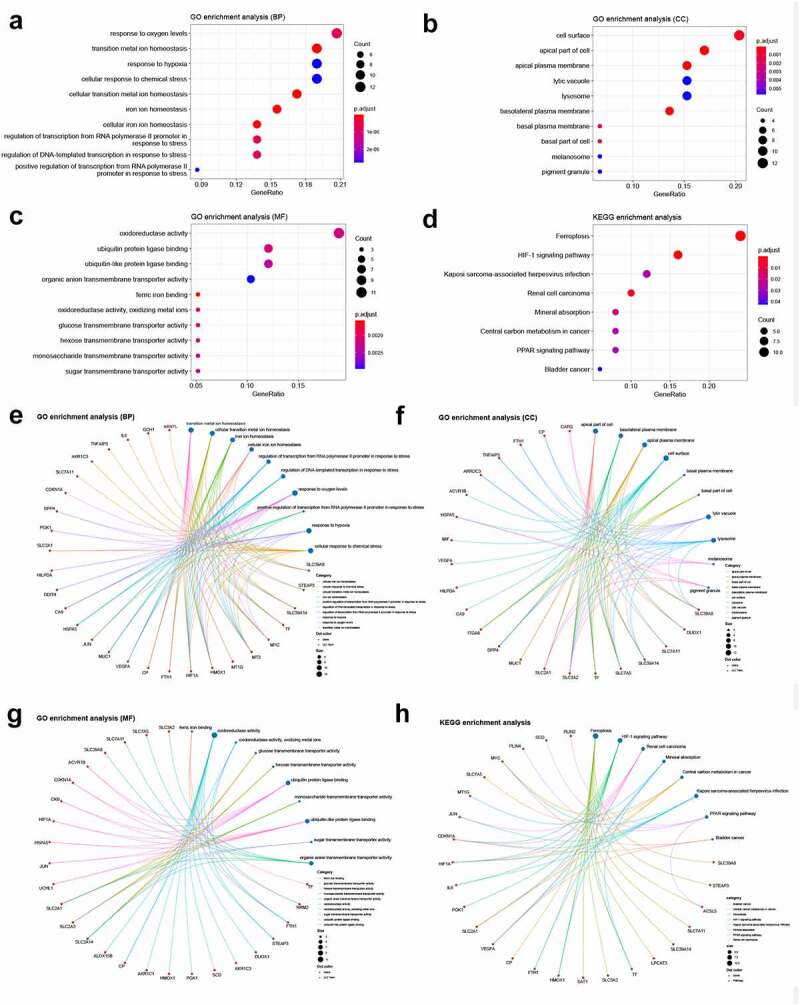
GO and KEGG enrichment analyses of differentially expressed ferroptosis-related genes

We conducted GO and KEGG enrichment analyses to understand the functions and related pathways of the DEFRGs. In the GO enrichment analysis, DEFRGs were mainly enriched in response to oxygen levels, transition metal ion homeostasis, and response to hypoxia in the biological process category (BP) (Figure 4a); cell surface, apical part of cell, and apical plasma membrane in the cellular component category (CC) (Figure 4b); and oxidoreductase activity, ubiquitin protein ligase binding, and ubiquitin-like protein ligase binding in the molecular function category (MF) (Figure 4c). In the KEGG enrichment analysis, most of the DEFRPs participated in the ferroptosis and HIF-1 signaling pathways (Figure 4d). We further analyzed the crosstalk between genes and different functions or pathways. The results suggested that the role of FRGs in the regulation of TAAD may be the result of the crosstalk of multiple gene functions and pathways, as shown in Figures 4e-h.

GO enrichment analysis of DEFRGs in (A) the biological process category (BP); (B) the cellular component category (CC); (C) the molecular function category (MF). (D) KEGG enrichment analysis of DEFRGs. Crosstalk analysis between DEFRGs and (E) gene functions in BP; (F) gene functions in CC; (G) gene functions in MF; (H) KEGG pathways. DEFRG, differentially expressed ferroptosis-related gene.

### PPI network and identification of key modules and hub genes

**Figure 5.**
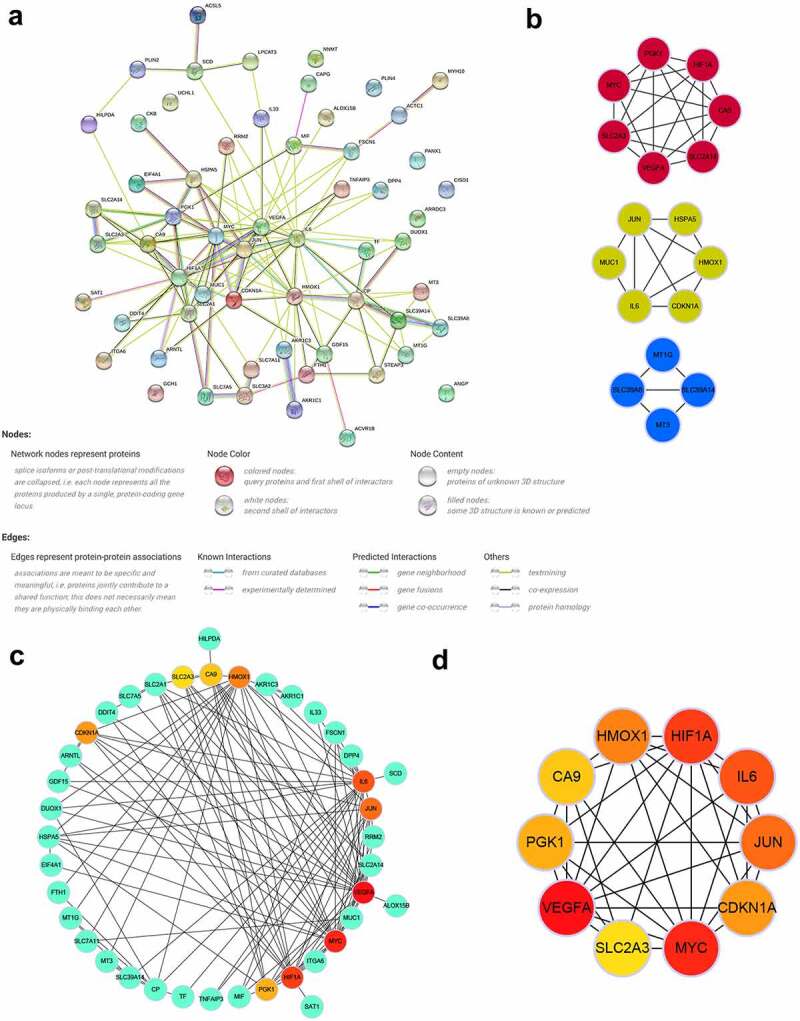
PPI network and identification of key modules and hub genes

To explore the interactions of these identified DEFRGs, we constructed a PPI network (Figure 5a) of DEFRGs using the STRING database. Cytoscape software was used to further analyze the data. Three key modules were identified using the MCODE plugin (Figure 5b), which may be important regulatory pathways for FRGs in TAAD development and. CA9, HMOX1, IL6, JUN, SLC2A3, CDKN1A, VEGFA, HIF1A, MYC, and PGK1 were selected as hub genes, which were the top 10 genes ranked by the MCC algorithm using the Cytohubba plugin. The multiple associations between hub genes and with other DEFRGs are shown in Figures 5c and d. The scores of all DEFRGs calculated by the Cytohubba plugin are listed in **Table S6**.

(A) PPI network of all DEFRGs constructed by STRING database. (B) three key modules identified by the Cytoscape plugin MCODE (the parameters were set to default: degree cutoff = 2, node score cutoff = 0.2, K-core = 2 and Max depth = 100). (C) Crosstalk between top 10 hub genes ranked by MCC algorithm and other DEFRGs. (D) Crosstalk between top 10 hub genes ranked by MCC algorithm, the deeper color of the dot means that the rank order of the hub gene is more advanced. DEFRG, differentially expressed ferroptosis-related gene.

### Validation of hub gene expression and predictive performance for TAAD in GSE52093

**Figure 6.**
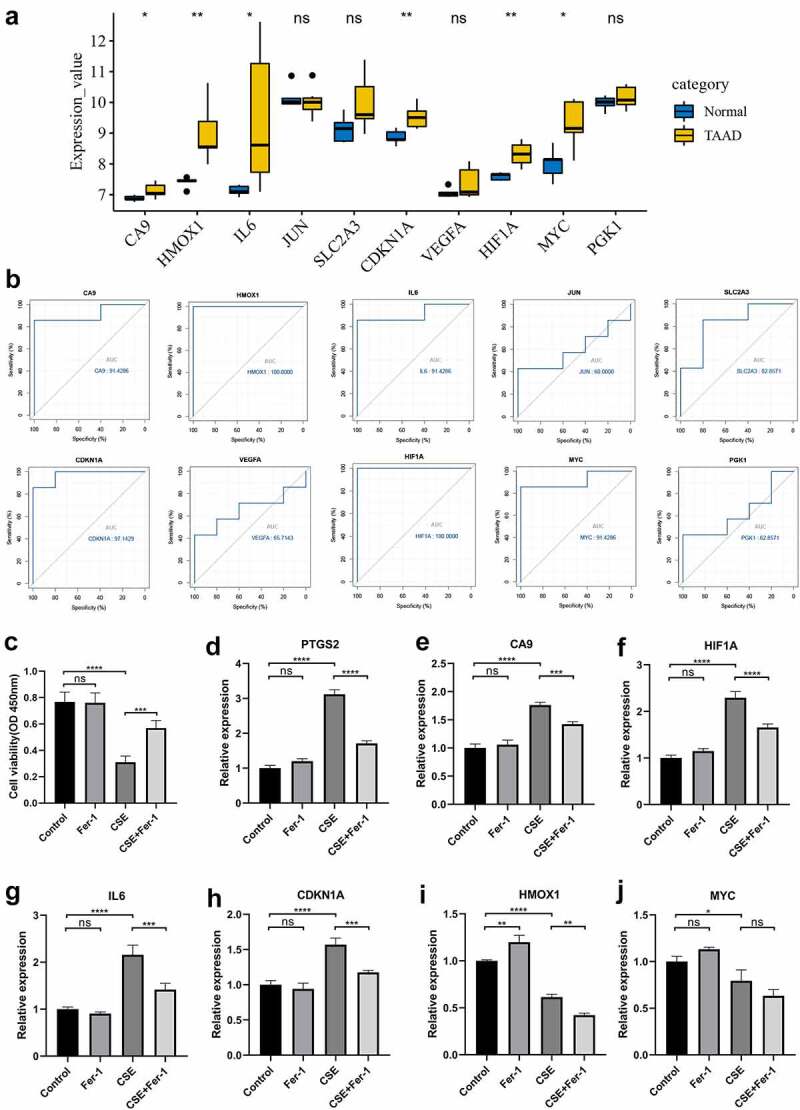
Validation of hub genes in GSE52093 and CSE-induced VSMC injury model

We chose to use another independent dataset, GSE52093, to further identify the key genes. After preprocessing the data from the GSE52093 dataset, the expression data of 10 selected hub genes were extracted and statistically analyzed. Six of the 10 hub genes (CA9, HMOX1, IL6, CDKN1A, HIF1A, and MYC) were significantly upregulated in the TAAD samples compared to normal samples (Figure 6a), consistent with the analysis in GSE153434. Analysis of ROC curves revealed that these six genes showed great predictive performance for TAAD in GSE52093 (Figure 6b), suggesting that these genes may be potential biomarkers for TAAD diagnosis. This may be particularly true for HIF1A and HMOX1 (AUC, 1.000). These genes were also included in the core genes identified by GSEA (**Figure S1**) and were ultimately identified as key genes.

(A) Expression levels of hub genes in TAAD and normal aortic samples in GSE52093. (B) Receiver operating characteristic (ROC) analysis showed the predictive performance of hub genes for TAAD in GSE52093 (AUC, area under the ROC curve). (C) A7r5 cells were treated with 100% cigarette smoke extract (CSE) for 4 h in the presence or absence of Fer-1 (5 μM), cell viability was assessed using the CCK8 assay (n = 3). (D-J) Changes in mRNA expression of hub genes after 4 h of 100%CSE treatment in the presence or absence of Fer-1 (5 μM) by qRT-PCR (n = 3). *p < 0.05, **p < 0.01, ***p < 0.001, ****p < 0.0001, ns = not significant.

### Validation of key gene expression in the CSE-induced VSMC injury model

VSMC injury is one of the major pathological mechanisms of TAAD, so we validated key genes in a model of CSE-induced A7r5 cell injury, a model that simulated smoking-induced VSMC injury in TAAD. Ferroptosis has been identified as an essential mechanism of CSE-induced injury in A7r5 cells, a type of rat thoracic artery smooth muscle cell, which has been demonstrated molecularly and morphologically, and the ferroptosis inhibitor Fer-1 can effectively protect A7r5 cells from CSE-induced injury by attenuating ferroptosis. We hypothesized that the key genes we identified might be involved in regulating CSE-induced ferroptosis in A7r5 cells, and we constructed a CSE-induced A7r5 cell injury model for validation. The CCK8 results were consistent with previous studies, and the viability of A7r5 cells was significantly reduced after 4 h of CSE treatment, which was greatly inhibited by Fer-1 (Figure 6c). The mRNA expression of the gene was examined by qRT-PCR, and the results showed that the ferroptosis-specific marker PTGS2 was significantly upregulated in A7r5 cells treated with CSE and that this PTGS2 upregulation was inhibited by Fer-1 (Figure 6d). Four of the six key genes, CA9, IL6, CDKN1A, and HIF1A, were significantly upregulated in CSE-treated A7r5 cells, and this upregulation was inhibited by Fer-1 (Figure 6e-h), consistent with the trends in the TAAD-related datasets. Interestingly, HMOX1 and MYC were downregulated in CSE-treated A7r5 cells (Figures 6i and j), which was contrary to the trends in datasets GSE153434 and GSE52093. These findings suggest that some of the key genes we identified may mediate smoking-induced TAAD development by regulating ferroptosis.

### Immune infiltration analyses

**Figure 7.**
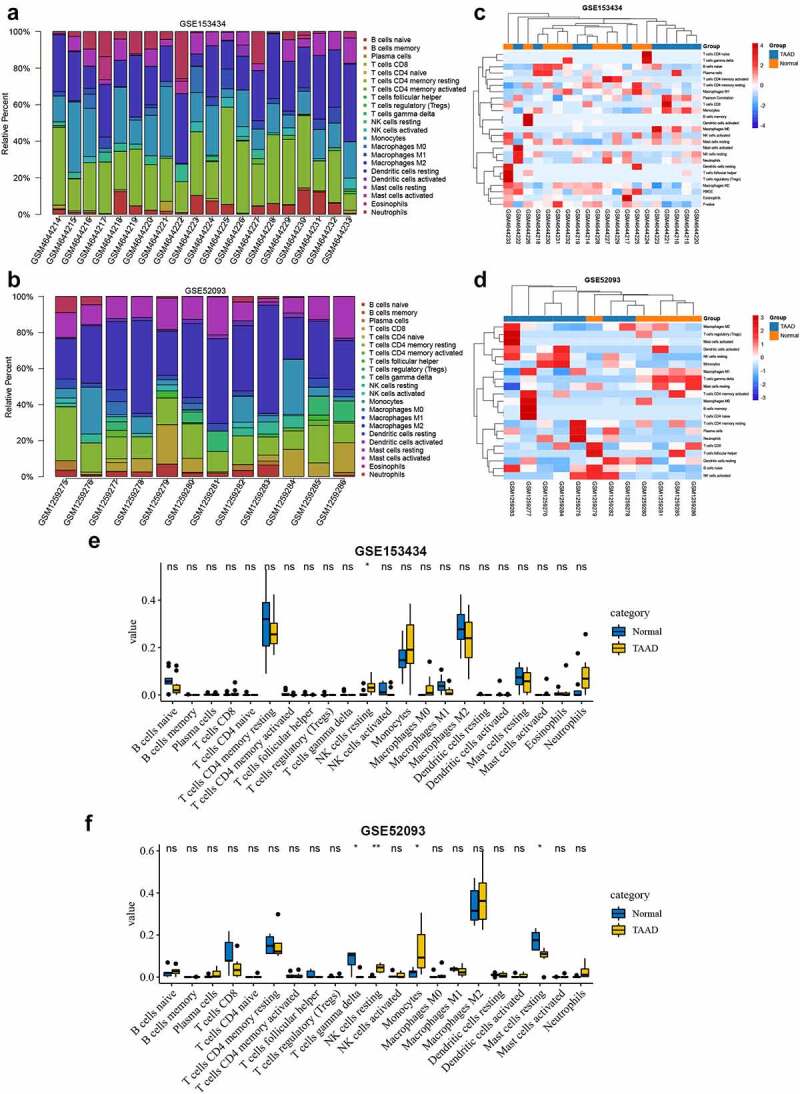
Immune infiltration analyses by the CIBERSORT algorithm in GSE153434 and GSE52093

Ferroptosis is one of the forms of immune cell death. Aberrant ferroptosis can affect the immune environment. Several key genes that we identified have been reported to be potentially involved in pathological immune environmental alterations, which may be associated with dysregulation of iron homeostasis [24,25]. Therefore, we performed an immune infiltration analysis to attempt to explore the crosstalk between ferroptosis and the immune response in TAAD. Figures 7a and b show the proportional histograms of the 22 immune cells in the GSE153434 and GSE52093 datasets calculated by the CIBERSORT algorithm. There were some differences in immune infiltration between the TAAD samples and normal samples, but the differences were not significant, consistent with the clustered heat map results (Figures 7c and d). Further statistical analysis showed that resting NK cells were significantly elevated in the TAAD samples in both the GSE153434 and GSE52093 datasets (Figures 7e and f), while the ssGSEA results showed that three immune cell types, Th17 cells, monocytes, and macrophages, were significantly elevated in the TAAD samples in both datasets (**Figure S2**). Interestingly, the infiltration scores calculated by ssGSEA in the TAAD samples were not significantly different from those in normal samples, either GSE153434 (p = 0.53) or GSE52093 (p = 0.11), suggesting that the role of immune infiltration on TAAD may be limited or have individual differences, and the regulation of ferroptosis in TAAD on immune infiltration requires further research.

the proportional histograms of the 22 immune cells calculated by the CIBERSORT algorithm in GSE153434 (A) and GSE52093 (B). Clustering heat map of immune cells in GSE153434 (C) and GSE52093 (D). Differences in the proportion of immune cells in TAAD and normal aortic samples in GSE153434 (E) and GSE52093 (F). Immune cells with a proportion of zero were hidden. *p < 0.05, **p < 0.01, ns = not significant.

## Discussion

Ferroptosis has been found to be involved in the development of various cardiovascular diseases, including ischemia/reperfusion myocardial injury, heart failure, heart transplantation, and doxorubicin-induced cardiotoxicity [11,12,26]. However, the relationship between ferroptosis and AD is still unclear. As a cardiovascular disease with high mortality, AD is attributed to the effects of multiple factors, such as genetic variants, diet, and environmental factors [1,18]. Cigarette smoking is one of the main risk factors for AD [16,17,27]. Cigarette extracts (acrolein and methyl vinyl ketone) could rapidly form conjugates with GSH and deplete GSH, inducing ferroptosis in a time and dose-dependent manner in vascular smooth muscle cells [15], while particulate matter 2.5 could induce ferroptosis in endothelial cells by triggering lipid peroxidation and increasing intracellular iron levels [28]. Given this, we speculate that ferroptosis may play an unrecognized role in AD. Thus, we performed a bioinformatic analysis in the TAAD-related datasets GSE153434 and GSE52093 to explore the regulatory influence and mechanism of ferroptosis on TAAD development.

Since the FRGs we selected included mRNAs, lncRNAs, and microRNAs, we chose the GSE153434 dataset which contains whole transcriptome data for analyses. The GSEA results showed a significant correlation between ferroptosis and TAAD, suggesting that ferroptosis is an important molecular characteristic in the development of TAAD. Further GO enrichment analysis revealed that DEFRGs may influence the response to oxygen levels, transition metal ion homeostasis, and the response to hypoxia by regulating oxidoreductase activity and post-transcriptional ubiquitination modifications, which may be the potential mechanism by which ferroptosis influences the development of TAAD. The KEGG enrichment analysis indicated that DEFRGs were mainly enriched in the ferroptosis and HIF-1 signaling pathways, suggesting that the HIF-1 signaling pathway, a critical pathway in the regulation of the body’s response to hypoxia, may play an important role in mediating ferroptosis in TAAD, which has also been proposed in other biological settings [29].

After constructing the PPI network, we selected three key modules and 10 hub genes. These modules may be important regulatory pathways for FRGs in TAAD development and could provide a reference for subsequent studies. Six of the 10 hub genes (CA9, HMOX1, IL6, CDKN1A, HIF1A, and MYC) were identified as differentially expressed and diagnostically significant in the GSE52093 dataset and were recognized as key genes involved in the role of ferroptosis in TAAD. All key genes have reliable evidence supporting their involvement in the regulation of ferroptosis in different biological circumstances [20,21], among which CA9, MYC, and CDKN1A function as ferroptosis suppressors [30–32] and IL6 functions as a ferroptosis promoter [33,34], while HMOX1 and HIF1A can function as both suppressors and promoters of ferroptosis [32,35–38]. Interestingly, in both datasets we studied, all key genes were upregulated in the TAAD samples, suggesting that the mechanisms of ferroptosis regulation in TAAD may be multiplicative and complicated. In addition, the key genes we identified are not ferroptosis-specific genes; they have been implicated in many other biological processes as well, and some of the key genes have been demonstrated to be regulators modulating TAAD development through mechanisms beyond ferroptosis [39–41]. Therefore, we performed further validation of the key genes in a model of CSE-induced VSMC injury, a model in which the important role of ferroptosis has been identified molecularly and morphologically [15]. Performing qRT-PCR, we determined that CA9, IL6, CDKN1A, and HIF1A were involved in the CSE-induced ferroptosis in VSMCs by regulating the mRNA expression levels of key genes, suggesting that they may mediate the development of smoking-related TAAD, and yet further studies are needed to clarify the specific regulatory mechanisms.

The relationship between different forms of cell death is complicated and poorly understood [42,43]. Although still unclear, several studies have demonstrated the intricate relationship between ferroptosis and autophagy [44–46]. A study found that autophagy participates in the development of TAAD and identified key genes regulating autophagy in TAAD [47]. We noticed that some genes in TAAD are involved in regulating both ferroptosis and autophagy pathways, suggesting potential crosstalk between ferroptosis and autophagy in the development of TAAD, especially in some selective autophagy processes [35].

Recent studies have shown that iron homeostasis plays an important role in the regulation of immune responses, and an imbalance in iron homeostasis may affect the development, function, and death of immune cells [48]. Ferroptosis has been identified as one of the forms of cell death in a variety of immune cells, affecting the immune response with potential interplay between ferroptosis and the immune response under certain circumstances [49]. The immune response plays a strong role in AD evocation and progression [27,50–52]. However, the immune infiltration analysis in our study showed no significant difference in immune infiltration between the normal and TAAD samples, suggesting that ferroptosis may regulate the development of TAAD through cells beyond immune cells, such as VSMCs. However, further studies are required for verification.

Given the lack of specific molecular markers for ferroptosis and the fact that many FRGs are also involved in various biological processes beyond ferroptosis, a combination of molecular biological and cellular morphological features to analyze the role played by ferroptosis in TAAD development is particularly critical for further studies. Further validation and biofunctional experiments in vivo are necessary but difficult. Current animal models of TAAD are mostly based on chemical induction, surgical procedures, or endovascular approaches, which morphologically reproduce human aortic dissection features. However, there are no available animal models that well reproduce the histological characteristics and natural history of TAAD, which makes validation potentially inaccurate [53–55]. In addition, there may be differences in the pathology of TAAD due to different etiologies, such as hypertension, aortitis, Marfan syndrome, etc. In this background, examination of protein expression levels and the morphological features of ferroptosis based on numerous human TAAD samples is urgently needed to deepen the understanding of the regulatory role of ferroptosis in the development of TAAD.

## Conclusion

We found differentially expressed ferroptosis-related genes in the normal and TAAD samples. These genes may influence the response to oxygen levels, transition metal ion homeostasis, and the response to hypoxia by regulating oxidoreductase activity and post-transcriptional ubiquitination modifications, which may be the potential functional mechanism of FRGs in TAAD development. The HIF-1 signaling pathway may be a key pathway regulating ferroptosis in TAAD development. Six ferroptosis-related key genes were identified and four of thems may be associated with the development of TAAD due to smoking. These findings will contribute to a better understanding of the unique role of ferroptosis in TAAD, but further studies are still required to clarify the complex regulatory mechanisms of ferroptosis in TAAD development.

## Supplementary Material

Supplemental Material

## Data Availability

Publicly available datasets were analyzed in this study. The data used to support the results of this study are available from the online website as mentioned above.
