# Supplementary material for: Role of ferroptosis-related genes in Stanford type a aortic dissection and identification of key genes: new insights from bioinformatic analysis
Source: Bioengineered. 2021 Nov 30;12(2):9976–90. doi: 10.1080/21655979.2021.1988840 (PMC8809966; doi:10.1080/21655979.2021.1988840)
Supplement: Supplemental Material [file KBIE_A_1988840_SM5399.zip › Supplementary Material.docx]

## Supplementary Material

Supplementary materials are available online at DOI: [10.6084/m9.figshare.14600631](https://doi.org/10.6084/m9.figshare.14600631). List as follow: **Figure S1**: The overlap of core genes and key genes; **Figure S2**: Immune infiltration analysis by ssGSEA; **Table S1**: The merged ferroptosis-related genes set; **Table S2**: Detailed primer information; **Table S3**: Core genes identified by GSEA in GSE153434; **Table S4**: DEGs identified in GSE153434; **Table S5**: DEFRGs identified in GSE153434; **Table S6**: Scores of all DEFRGs calculated by the Cytohubba plugin.
