# Supplementary figures and images for: Role of ferroptosis-related genes in Stanford type a aortic dissection and identification of key genes: new insights from bioinformatic analysis
Source: Bioengineered. 2021 Nov 30;12(2):9976–90. doi: 10.1080/21655979.2021.1988840 (PMC8809966; doi:10.1080/21655979.2021.1988840)

**Figure S1 The overlap of core genes and key genes**

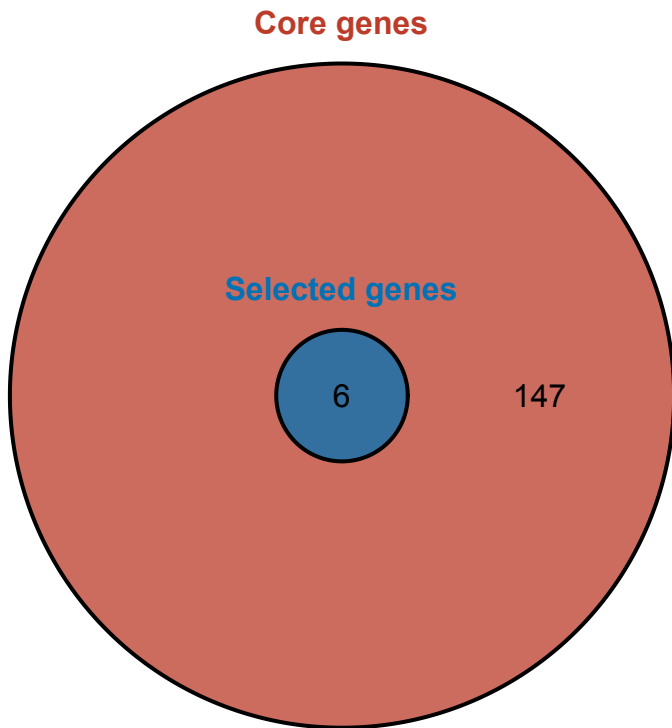

Supplement: Supplemental Material [file KBIE_A_1988840_SM5399.zip › Fig S1.pdf]

# Figure S2 Immune infiltration analysis by ssGSEA

**A**

**GSE153434**

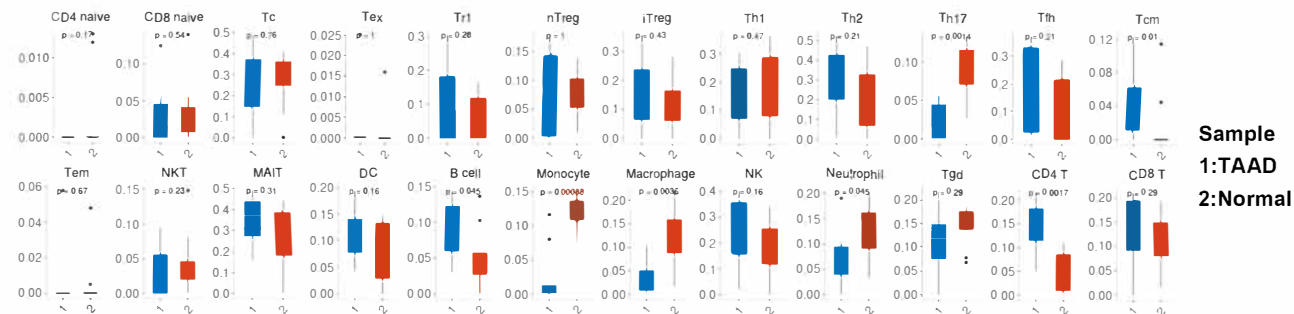

**B**

**GSE52093**

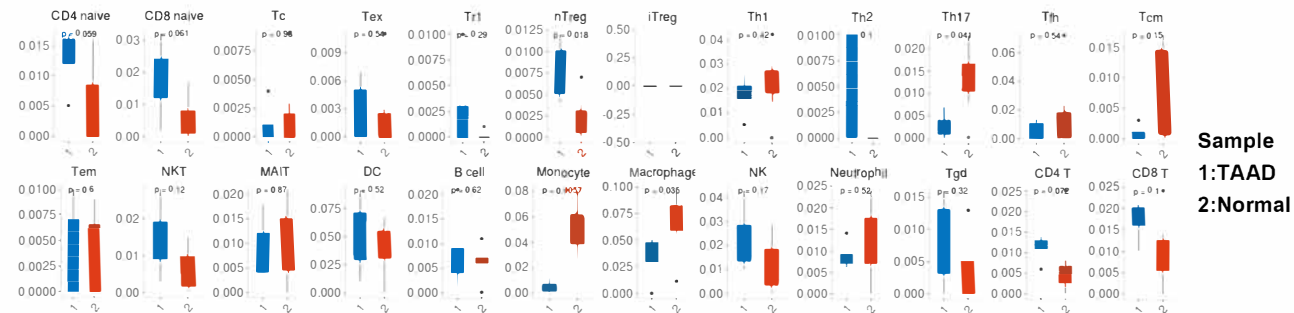

Supplement: Supplemental Material [file KBIE_A_1988840_SM5399.zip › Fig S2.pdf]
